# Tetra-μ-acetato-κ^8^ *O*:*O*′-bis­{[2-(2-fur­yl)-1-(2-furylmeth­yl)-1*H*-benzimidazole-κ*N* ^3^]copper(II)}

**DOI:** 10.1107/S1600536809025276

**Published:** 2009-07-04

**Authors:** Qiang Wang, Cai-Feng Bi, Da-Qi Wang, Yu-Hua Fan, Ji-Tian Liu

**Affiliations:** a College of Chemistry and Chemical Engineering, Ocean University of China, Shandong 266100, People’s Republic of China; b College of Chemistry and Chemical Engineering, Liaocheng University, Shandong 252059, People’s Republic of China

## Abstract

The title complex, [Cu_2_(CH_3_COO)_4_(C_16_H_12_N_2_O_2_)_2_], forms a dimer of the paddle-wheel type located on a crystallographic inversion centre. The two Cu^II^ atoms [Cu⋯Cu = 2.7254 (11) Å] are bridged by four acetate anions. The geometry of the polyhedron around the metal centre can be described as tetra­gonal-pyramidal derived from the calculation of the value τ = 0.0018. The apical positions of the tetra­gonal-pyramidal copper coordination polyhedra are occupied by the N atoms of 2-(2-fur­yl)-1-(2-furylmeth­yl)-1*H*-benzimidazole ligands. In the crystal structure, mol­ecules are linked into a chain by inter­molecular C—H⋯O hydrogen bonds parallel to [010]. Two furan rings are disordered over two positions in ratios of 0.55:0.45 and 0.69:0.31.

## Related literature

For general background, see: Solomon *et al.* (1992 ▶). For the chemical, physical and structural properties of tripodal copper complexes, see: Malachowski *et al.* (1995 ▶); Mclachlan *et al.* (1995 ▶). For Cu—Cu distances in dimeric copper(II) carboxyl­ate complexes, see: Liu *et al.* (2007 ▶). For the τ parameter, see: Addison *et al.* (1984 ▶). 
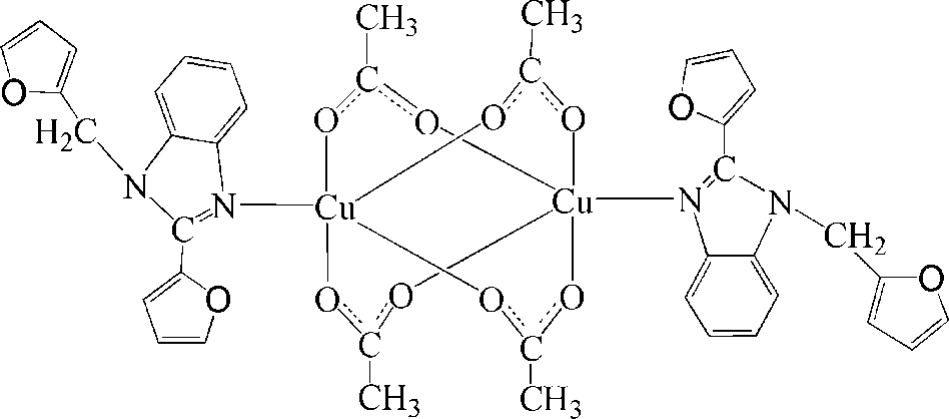


## Experimental

### 

#### Crystal data

[Cu_2_(C_2_H_3_O_2_)_4_(C_16_H_12_N_2_O_2_)_2_]
*M* *_r_* = 891.81
Triclinic, 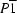

*a* = 9.6617 (15) Å
*b* = 11.2779 (19) Å
*c* = 19.233 (2) Å
α = 75.817 (1)°
β = 80.031 (2)°
γ = 76.168 (1)°
*V* = 1958.3 (5) Å^3^
*Z* = 2
Mo *K*α radiation
μ = 1.16 mm^−1^
*T* = 298 K
0.48 × 0.23 × 0.12 mm

#### Data collection

Siemens SMART CCD area-detector diffractometer
Absorption correction: multi-scan (*SADABS*; Sheldrick, 1996 ▶) *T*_min_ = 0.607, *T*_max_ = 0.874
10261 measured reflections
6802 independent reflections
4761 reflections with *I* > 2σ(*I*)
*R*_int_ = 0.023

#### Refinement

*R*[*F* ^2^ > 2σ(*F* ^2^)] = 0.057
*wR*(*F* ^2^) = 0.152
*S* = 1.02
6802 reflections
606 parameters
H-atom parameters constrained
Δρ_max_ = 0.59 e Å^−3^
Δρ_min_ = −0.62 e Å^−3^

### 

Data collection: *SMART* (Siemens, 1996 ▶); cell refinement: *SAINT* (Siemens, 1996 ▶); data reduction: *SAINT*; program(s) used to solve structure: *SHELXS97* (Sheldrick, 2008 ▶); program(s) used to refine structure: *SHELXL97* (Sheldrick, 2008 ▶); molecular graphics: *SHELXTL* (Sheldrick, 2008 ▶); software used to prepare material for publication: *SHELXTL*.

## Supplementary Material

Crystal structure: contains datablocks I, global. DOI: 10.1107/S1600536809025276/bv2119sup1.cif

Structure factors: contains datablocks I. DOI: 10.1107/S1600536809025276/bv2119Isup2.hkl

Additional supplementary materials:  crystallographic information; 3D view; checkCIF report

## Figures and Tables

**Table:** 

Cu1—O4^i^	1.961 (3)
Cu1—O3	1.966 (3)
Cu1—O5	1.977 (3)
Cu1—O6^i^	1.989 (3)
Cu1—N1	2.233 (4)

**Table:** 

O4^i^—Cu1—O3	165.00 (14)
O5—Cu1—O6^i^	165.11 (15)

Symmetry code: (i) 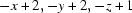.

**Table 2:** Hydrogen-bond geometry (Å, °)

*D*—H⋯*A*	*D*—H	H⋯*A*	*D*⋯*A*	*D*—H⋯*A*
C12—H12*B*⋯O3^ii^	0.97	2.55	3.426 (7)	151
C31—H31⋯O6^iii^	0.93	2.54	3.44 (3)	166
C36—H36⋯O4^i^	0.93	2.50	3.371 (19)	157

Symmetry codes: (i) 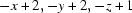; (ii) 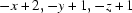; (iii) 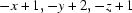.

**Fig. 1.**
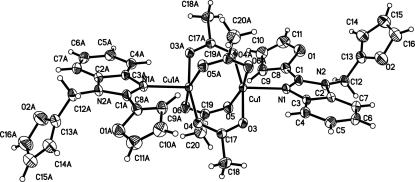
The structure of the title complex, showing 30% probability displacement ellipsoids and the atom-numbering scheme.

**Fig. 2.**
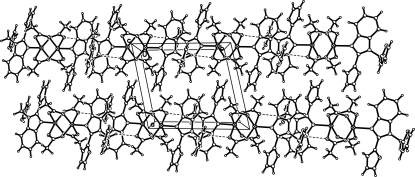
The crystal packing of the title complex, viewed approximately along the b axis.

## supplementary crystallographic information

### Comment

Investigations of the coordination chemistry of copper(II) continue to be stimulated by interest in developing models for copper proteins and in understanding the factors which give rise to the seemingly infinite variety of distortions from regular stereochemistry observed in Cu^II^ complexes (Solomon *et al.*, 1992). Due to the unique coordination polyhedra and their ease of preparation, tripodal copper complexes have attracted much attention in addition to their special chemical, physical and structure properties (Malachowski *et al.*, 1995; Mclachlan *et al.*, 1995). We report here the synthesis and crystal structure of the title compound, a new binuclear copper(II) complex.

The molecular structure of the title complex is shown in Fig.1. The Cu^II^ atom is five-coordinated, with a coordination geometry that is best described as distorted square pyramidal. The Cu1—Cu1A distance is 2.7254 (11) Å, very similar to the values found in other dimeric copper(II) carboxylate complexes (Liu *et al.*, 2007). The basal plane for a tetragonal-pyramidal geometry is defined by the atoms O3, O4, O5 and O6, their mean deviation from this plane is 0.0031 Å, and the Cu atom just out of this plane by 0.4762 Å. The axial position of the pyramid is occupied by atom N1. For this point of view, a geometry parameter τ, which is defined τ = (β - α)/60, applicable to 5-coordinate structures within the structural continuum between trigonal bipyramidal and tetragonal or rectangular pyramidal. For a perfect tetragonal symmetry τ is zero, and for a perfect trigonal-bipyramidal geometry τ becomes 1.0 (Addison *et al.* 1984). In the title compound, the largest angles within the four atoms O3, O4, O5, O6, are β = 165.11 (15)° for O5–Cu1–O6, and α = 165.00 (14)° for O3–Cu1–O4. Thus, τ is (165.11–165.00)/60 = 0.0018, indicating a 98% rectangular pyramidal geometry. The 2-(furan-2-yl)-1-((furan-2-yl)methyl) -1*H*-benzo[*d*]imidazole molecules are coordinated to Cu^II^ through their midazole N atom and occupy the axial position. Four acetate ligands act as bridges to connect the two Cu^II^ centers into a dinuclear complex across a crystallographic centre of inversion. In the ligand, the dihedral angle between furyl rings and phenyl ring are 24.50 (3)°, 55.22 (2)° and 30.84 (2) °. Selected bond distances and angles are presented in Table 1.

As seen in Fig. 2, the molecules are linked into a one-dimensional chain by intermolecular C—H···O hydrogen bonds. (Table 2).

### Experimental

furfuraldehyde (10 mmol, 960 mg) was added dropwise to a absolute ethanol (20 ml) of *o*-phenylenediamine (5 mmol, 547 mg). The mixture was heated under reflux with stirring for 2 h. An absolute ethanol solution (10 ml) of cupric acetate monohydrate (5 mmol, 850 mg) was then added dropwise, and the mixture was stirred at room temperature for another 20 h. The solution was filtered off, the filterate was kept at room temperature for about several weeks, after which the green crystals were obtained.

### Refinement

All H-atoms were positioned geometrically and refined using a riding model, with C—H = 0.93–0.96 Å, with *U*_iso_(H) =1.2*U*_eq_(C).

### Crystal data

**Table:**

[Cu_2_(C_2_H_3_O_2_)_4_(C_16_H_12_N_2_O_2_)_2_]	*Z* = 2
*M**_r_* = 891.81	*F*(000) = 916
Triclinic, *P*1	*D*_x_ = 1.512 Mg m^−^^3^
Hall symbol: -P 1	Mo *K*α radiation, λ = 0.71073 Å
*a* = 9.6617 (15) Å	Cell parameters from 2908 reflections
*b* = 11.2779 (19) Å	θ = 2.4–24.7°
*c* = 19.233 (2) Å	µ = 1.16 mm^−^^1^
α = 75.817 (1)°	*T* = 298 K
β = 80.031 (2)°	Block, green
γ = 76.168 (1)°	0.48 × 0.23 × 0.12 mm
*V* = 1958.3 (5) Å^3^	

### Data collection

**Table:**

Siemens SMART CCD area-detector diffractometer	6802 independent reflections
Radiation source: fine-focus sealed tube	4761 reflections with *I* > 2σ(*I*)
graphite	*R*_int_ = 0.023
φ and ω scans	θ_max_ = 25.0°, θ_min_ = 1.9°
Absorption correction: multi-scan (*SADABS*; Sheldrick, 1996)	*h* = −11→11
*T*_min_ = 0.607, *T*_max_ = 0.874	*k* = −13→13
10261 measured reflections	*l* = −17→22

### Refinement

**Table:**

Refinement on *F*^2^	Primary atom site location: structure-invariant direct methods
Least-squares matrix: full	Secondary atom site location: difference Fourier map
*R*[*F*^2^ > 2σ(*F*^2^)] = 0.057	Hydrogen site location: inferred from neighbouring sites
*wR*(*F*^2^) = 0.152	H-atom parameters constrained
*S* = 1.02	*w* = 1/[σ^2^(*F*_o_^2^) + (0.0617*P*)^2^ + 4.0885*P*] where *P* = (*F*_o_^2^ + 2*F*_c_^2^)/3
6802 reflections	(Δ/σ)_max_ = 0.001
606 parameters	Δρ_max_ = 0.59 e Å^−^^3^
0 restraints	Δρ_min_ = −0.62 e Å^−^^3^

### Special details

**Table:**

Geometry. All e.s.d.'s (except the e.s.d. in the dihedral angle between two l.s. planes) are estimated using the full covariance matrix. The cell e.s.d.'s are taken into account individually in the estimation of e.s.d.'s in distances, angles and torsion angles; correlations between e.s.d.'s in cell parameters are only used when they are defined by crystal symmetry. An approximate (isotropic) treatment of cell e.s.d.'s is used for estimating e.s.d.'s involving l.s. planes.
Refinement. Refinement of *F*^2^ against ALL reflections. The weighted *R*-factor *wR* and goodness of fit *S* are based on *F*^2^, conventional *R*-factors *R* are based on *F*, with *F* set to zero for negative *F*^2^. The threshold expression of *F*^2^ > σ(*F*^2^) is used only for calculating *R*-factors(gt) *etc*. and is not relevant to the choice of reflections for refinement. *R*-factors based on *F*^2^ are statistically about twice as large as those based on *F*, and *R*- factors based on ALL data will be even larger.

### Fractional atomic coordinates and isotropic or equivalent isotropic displacement parameters (Å^2^)

**Table:**

	*x*	*y*	*z*	*U*_iso_*/*U*_eq_	Occ. (<1)
Cu1	0.99281 (6)	0.89727 (5)	0.47651 (3)	0.03232 (18)	
Cu2	0.53213 (7)	0.60430 (6)	0.01052 (3)	0.0440 (2)	
N1	0.9845 (4)	0.7264 (4)	0.4396 (2)	0.0365 (9)	
N2	0.9293 (5)	0.5746 (4)	0.3999 (2)	0.0436 (10)	
N3	0.5831 (5)	0.7740 (4)	0.0330 (3)	0.0551 (12)	
N4	0.6085 (6)	0.9327 (5)	0.0755 (3)	0.0654 (14)	
O1	0.6522 (4)	0.7372 (4)	0.3768 (2)	0.0650 (11)	
O2	0.9699 (6)	0.4443 (5)	0.2714 (3)	0.0931 (16)	
O3	1.1457 (4)	0.8153 (3)	0.53831 (19)	0.0480 (9)	
O4	1.1553 (4)	0.9823 (3)	0.57688 (19)	0.0471 (9)	
O5	0.8573 (4)	0.8762 (3)	0.56612 (19)	0.0487 (9)	
O6	0.8639 (4)	1.0444 (3)	0.60436 (19)	0.0507 (9)	
O7	0.3864 (18)	0.8787 (16)	0.1965 (9)	0.072 (5)	0.55 (3)
O8	0.7106 (13)	1.0211 (14)	0.2262 (8)	0.083 (4)	0.69 (3)
O7'	0.3368 (18)	0.7504 (16)	0.1357 (12)	0.076 (6)	0.45 (3)
O8'	0.752 (3)	1.082 (3)	0.1872 (18)	0.076 (9)	0.31 (3)
O9	0.7334 (4)	0.5180 (4)	−0.0055 (2)	0.0673 (12)	
O10	0.6774 (5)	0.3442 (4)	−0.0174 (2)	0.0682 (12)	
O11	0.5353 (5)	0.6649 (4)	−0.0946 (2)	0.0626 (11)	
O12	0.4755 (5)	0.4963 (4)	−0.1098 (2)	0.0628 (11)	
C1	0.8807 (6)	0.6855 (5)	0.4222 (3)	0.0423 (12)	
C2	1.0751 (6)	0.5432 (5)	0.4036 (3)	0.0430 (12)	
C3	1.1091 (5)	0.6365 (4)	0.4280 (3)	0.0369 (11)	
C4	1.2510 (5)	0.6321 (5)	0.4367 (3)	0.0462 (13)	
H4	1.2761	0.6946	0.4527	0.055*	
C5	1.3516 (6)	0.5306 (5)	0.4203 (3)	0.0541 (14)	
H5	1.4471	0.5242	0.4256	0.065*	
C6	1.3142 (7)	0.4375 (5)	0.3961 (3)	0.0574 (16)	
H6	1.3859	0.3708	0.3855	0.069*	
C7	1.1777 (6)	0.4401 (5)	0.3875 (3)	0.0522 (14)	
H7	1.1534	0.3770	0.3717	0.063*	
C8	0.7319 (6)	0.7505 (5)	0.4262 (3)	0.0463 (13)	
C9	0.6530 (6)	0.8268 (5)	0.4672 (3)	0.0509 (14)	
H9	0.6820	0.8506	0.5045	0.061*	
C10	0.5158 (6)	0.8650 (6)	0.4429 (4)	0.0658 (17)	
H10	0.4369	0.9183	0.4616	0.079*	
C11	0.5204 (7)	0.8118 (7)	0.3896 (4)	0.0720 (19)	
H11	0.4441	0.8231	0.3634	0.086*	
C12	0.8467 (6)	0.4934 (5)	0.3834 (3)	0.0528 (14)	
H12A	0.7483	0.5143	0.4054	0.063*	
H12B	0.8857	0.4075	0.4056	0.063*	
C13	0.8457 (7)	0.5017 (6)	0.3052 (3)	0.0614 (16)	
C14	0.7492 (8)	0.5439 (7)	0.2608 (4)	0.077 (2)	
H14	0.6554	0.5856	0.2717	0.093*	
C15	0.8107 (10)	0.5159 (8)	0.1935 (4)	0.087 (2)	
H15	0.7661	0.5360	0.1518	0.104*	
C16	0.9412 (10)	0.4566 (8)	0.2008 (4)	0.097 (3)	
H16	1.0066	0.4264	0.1645	0.117*	
C17	1.1925 (5)	0.8705 (5)	0.5751 (3)	0.0408 (12)	
C18	1.3045 (7)	0.7915 (6)	0.6228 (4)	0.0682 (18)	
H18A	1.3664	0.8424	0.6288	0.102*	
H18B	1.3603	0.7248	0.6007	0.102*	
H18C	1.2579	0.7572	0.6691	0.102*	
C19	0.8264 (5)	0.9439 (5)	0.6114 (3)	0.0424 (12)	
C20	0.7392 (7)	0.8957 (6)	0.6811 (3)	0.0714 (19)	
H20A	0.7889	0.8145	0.7035	0.107*	
H20B	0.6473	0.8898	0.6712	0.107*	
H20C	0.7259	0.9520	0.7131	0.107*	
C21	0.5397 (7)	0.8363 (6)	0.0854 (4)	0.0631 (17)	
C22	0.7013 (7)	0.9309 (6)	0.0141 (4)	0.0654 (17)	
C23	0.8006 (8)	1.0051 (6)	−0.0221 (4)	0.076 (2)	
H23	0.8120	1.0710	−0.0041	0.091*	
C24	0.8779 (8)	0.9787 (7)	−0.0827 (5)	0.082 (2)	
H24	0.9444	1.0271	−0.1067	0.098*	
C25	0.8630 (8)	0.8812 (7)	−0.1118 (4)	0.079 (2)	
H25	0.9182	0.8668	−0.1547	0.094*	
C26	0.7671 (7)	0.8059 (6)	−0.0773 (4)	0.0671 (18)	
H26	0.7563	0.7406	−0.0961	0.081*	
C27	0.6876 (7)	0.8316 (5)	−0.0134 (4)	0.0596 (16)	
C28	0.4355 (8)	0.8056 (6)	0.1463 (4)	0.0667 (17)	
C29	0.371 (2)	0.705 (2)	0.1747 (15)	0.078 (5)	0.55 (3)
H29	0.3907	0.6345	0.1548	0.093*	0.55 (3)
C30	0.275 (3)	0.719 (3)	0.2356 (16)	0.073 (6)	0.55 (3)
H30	0.2177	0.6646	0.2630	0.088*	0.55 (3)
C31	0.285 (3)	0.832 (3)	0.2461 (15)	0.072 (6)	0.55 (3)
H31	0.2300	0.8709	0.2820	0.086*	0.55 (3)
C29'	0.421 (3)	0.819 (3)	0.2146 (16)	0.069 (6)	0.45 (3)
H29'	0.4782	0.8546	0.2346	0.083*	0.45 (3)
C30'	0.306 (4)	0.772 (3)	0.2482 (18)	0.073 (8)	0.45 (3)
H30'	0.2689	0.7660	0.2966	0.088*	0.45 (3)
C31'	0.255 (3)	0.735 (2)	0.198 (2)	0.077 (7)	0.45 (3)
H31'	0.1723	0.7018	0.2069	0.093*	0.45 (3)
C32	0.5972 (8)	1.0210 (6)	0.1213 (4)	0.0725 (19)	
H32A	0.5005	1.0383	0.1460	0.087*	0.69 (3)
H32B	0.6202	1.0989	0.0926	0.087*	0.69 (3)
H32C	0.5067	1.0239	0.1526	0.087*	0.31 (3)
H32D	0.5950	1.1036	0.0908	0.087*	0.31 (3)
C33	0.703 (11)	0.960 (6)	0.175 (5)	0.069 (9)	0.69 (3)
C34	0.808 (5)	0.854 (3)	0.1808 (18)	0.070 (6)	0.69 (3)
H34	0.8277	0.7962	0.1513	0.085*	0.69 (3)
C35	0.879 (3)	0.848 (2)	0.2387 (14)	0.072 (5)	0.69 (3)
H35	0.9523	0.7839	0.2571	0.086*	0.69 (3)
C36	0.821 (2)	0.956 (2)	0.2633 (13)	0.071 (5)	0.69 (3)
H36	0.8522	0.9797	0.2999	0.086*	0.69 (3)
C33'	0.72 (2)	0.992 (14)	0.166 (12)	0.07 (2)	0.31 (3)
C34'	0.783 (10)	0.881 (7)	0.200 (4)	0.073 (13)	0.31 (3)
H34'	0.7785	0.8034	0.1935	0.088*	0.31 (3)
C35'	0.861 (5)	0.903 (5)	0.246 (3)	0.067 (13)	0.31 (3)
H35'	0.9158	0.8440	0.2786	0.081*	0.31 (3)
C36'	0.842 (3)	1.028 (4)	0.235 (2)	0.074 (10)	0.31 (3)
H36'	0.8867	1.0695	0.2583	0.089*	0.31 (3)
C37	0.7653 (7)	0.4081 (6)	−0.0139 (4)	0.0640 (17)	
C38	0.9219 (7)	0.3457 (7)	−0.0194 (5)	0.099 (3)	
H38A	0.9787	0.4047	−0.0191	0.149*	
H38B	0.9488	0.3159	−0.0635	0.149*	
H38C	0.9380	0.2766	0.0210	0.149*	
C39	0.5055 (7)	0.6021 (6)	−0.1320 (3)	0.0583 (15)	
C40	0.5108 (9)	0.6544 (8)	−0.2121 (3)	0.093 (3)	
H40A	0.5917	0.6069	−0.2369	0.140*	
H40B	0.5199	0.7401	−0.2223	0.140*	
H40C	0.4240	0.6497	−0.2283	0.140*	

### Atomic displacement parameters (Å^2^)

**Table:**

	*U*^11^	*U*^22^	*U*^33^	*U*^12^	*U*^13^	*U*^23^
Cu1	0.0369 (3)	0.0277 (3)	0.0352 (3)	−0.0056 (2)	−0.0076 (2)	−0.0107 (2)
Cu2	0.0564 (4)	0.0363 (4)	0.0446 (4)	−0.0141 (3)	−0.0139 (3)	−0.0089 (3)
N1	0.038 (2)	0.037 (2)	0.039 (2)	−0.0074 (18)	−0.0056 (18)	−0.0167 (18)
N2	0.055 (3)	0.039 (2)	0.045 (2)	−0.014 (2)	−0.009 (2)	−0.017 (2)
N3	0.071 (3)	0.042 (3)	0.064 (3)	−0.016 (2)	−0.025 (3)	−0.016 (2)
N4	0.086 (4)	0.050 (3)	0.073 (4)	−0.013 (3)	−0.035 (3)	−0.021 (3)
O1	0.063 (3)	0.071 (3)	0.070 (3)	−0.015 (2)	−0.020 (2)	−0.022 (2)
O2	0.107 (4)	0.104 (4)	0.070 (3)	−0.001 (3)	−0.024 (3)	−0.034 (3)
O3	0.054 (2)	0.039 (2)	0.054 (2)	0.0039 (17)	−0.0209 (18)	−0.0181 (17)
O4	0.057 (2)	0.037 (2)	0.052 (2)	−0.0056 (17)	−0.0248 (18)	−0.0101 (17)
O5	0.054 (2)	0.051 (2)	0.047 (2)	−0.0209 (18)	0.0022 (18)	−0.0164 (18)
O6	0.058 (2)	0.047 (2)	0.047 (2)	−0.0133 (18)	0.0108 (18)	−0.0192 (18)
O7	0.091 (9)	0.061 (9)	0.073 (9)	−0.011 (7)	−0.019 (6)	−0.029 (7)
O8	0.106 (7)	0.072 (7)	0.079 (8)	−0.013 (6)	−0.026 (6)	−0.028 (6)
O7'	0.094 (10)	0.061 (9)	0.076 (12)	−0.003 (7)	−0.023 (8)	−0.023 (8)
O8'	0.094 (14)	0.068 (15)	0.078 (17)	−0.016 (11)	−0.026 (12)	−0.026 (14)
O9	0.065 (3)	0.052 (3)	0.083 (3)	−0.014 (2)	−0.011 (2)	−0.007 (2)
O10	0.065 (3)	0.057 (3)	0.085 (3)	−0.006 (2)	−0.014 (2)	−0.023 (2)
O11	0.084 (3)	0.057 (3)	0.056 (2)	−0.032 (2)	−0.022 (2)	−0.003 (2)
O12	0.087 (3)	0.066 (3)	0.045 (2)	−0.030 (2)	−0.020 (2)	−0.007 (2)
C1	0.050 (3)	0.039 (3)	0.045 (3)	−0.015 (2)	−0.007 (2)	−0.016 (2)
C2	0.053 (3)	0.039 (3)	0.038 (3)	−0.005 (2)	−0.008 (2)	−0.015 (2)
C3	0.040 (3)	0.036 (3)	0.037 (3)	−0.005 (2)	−0.004 (2)	−0.014 (2)
C4	0.045 (3)	0.047 (3)	0.048 (3)	−0.003 (2)	−0.007 (2)	−0.017 (3)
C5	0.047 (3)	0.056 (4)	0.055 (3)	0.004 (3)	−0.010 (3)	−0.016 (3)
C6	0.063 (4)	0.046 (3)	0.056 (4)	0.011 (3)	−0.005 (3)	−0.020 (3)
C7	0.062 (4)	0.043 (3)	0.052 (3)	0.000 (3)	−0.008 (3)	−0.020 (3)
C8	0.046 (3)	0.050 (3)	0.053 (3)	−0.020 (3)	−0.015 (3)	−0.015 (3)
C9	0.047 (3)	0.051 (3)	0.062 (4)	−0.015 (3)	−0.006 (3)	−0.021 (3)
C10	0.044 (3)	0.066 (4)	0.084 (5)	−0.007 (3)	−0.002 (3)	−0.018 (4)
C11	0.049 (4)	0.081 (5)	0.087 (5)	−0.011 (3)	−0.023 (4)	−0.013 (4)
C12	0.068 (4)	0.049 (3)	0.054 (3)	−0.022 (3)	−0.014 (3)	−0.020 (3)
C13	0.077 (4)	0.058 (4)	0.060 (4)	−0.019 (3)	−0.017 (4)	−0.023 (3)
C14	0.088 (5)	0.079 (5)	0.073 (5)	−0.015 (4)	−0.026 (4)	−0.022 (4)
C15	0.106 (6)	0.091 (6)	0.073 (5)	−0.018 (5)	−0.031 (5)	−0.023 (4)
C16	0.119 (7)	0.106 (7)	0.068 (5)	−0.002 (6)	−0.018 (5)	−0.037 (5)
C17	0.040 (3)	0.040 (3)	0.043 (3)	−0.005 (2)	−0.009 (2)	−0.010 (2)
C18	0.067 (4)	0.056 (4)	0.082 (5)	0.006 (3)	−0.041 (4)	−0.011 (3)
C19	0.043 (3)	0.040 (3)	0.043 (3)	−0.008 (2)	−0.002 (2)	−0.009 (2)
C20	0.081 (5)	0.070 (4)	0.054 (4)	−0.020 (4)	0.021 (3)	−0.012 (3)
C21	0.080 (4)	0.050 (4)	0.071 (4)	−0.013 (3)	−0.032 (4)	−0.018 (3)
C22	0.081 (5)	0.050 (4)	0.076 (5)	−0.020 (3)	−0.036 (4)	−0.008 (3)
C23	0.090 (5)	0.060 (4)	0.088 (5)	−0.027 (4)	−0.034 (4)	−0.008 (4)
C24	0.089 (5)	0.066 (5)	0.093 (6)	−0.035 (4)	−0.026 (5)	0.006 (4)
C25	0.085 (5)	0.070 (5)	0.082 (5)	−0.028 (4)	−0.024 (4)	0.003 (4)
C26	0.079 (5)	0.056 (4)	0.073 (5)	−0.024 (3)	−0.025 (4)	−0.005 (3)
C27	0.073 (4)	0.046 (3)	0.070 (4)	−0.020 (3)	−0.031 (4)	−0.009 (3)
C28	0.085 (5)	0.054 (4)	0.068 (5)	−0.003 (4)	−0.026 (4)	−0.024 (4)
C29	0.098 (14)	0.058 (10)	0.076 (14)	−0.004 (9)	−0.016 (11)	−0.021 (10)
C30	0.095 (15)	0.061 (15)	0.068 (16)	−0.007 (10)	−0.018 (12)	−0.023 (12)
C31	0.090 (14)	0.059 (15)	0.072 (11)	−0.005 (13)	−0.020 (9)	−0.025 (13)
C29'	0.085 (16)	0.060 (15)	0.071 (14)	−0.012 (13)	−0.026 (11)	−0.021 (12)
C30'	0.091 (19)	0.06 (2)	0.072 (14)	−0.009 (18)	−0.018 (12)	−0.020 (19)
C31'	0.088 (17)	0.064 (12)	0.08 (2)	−0.007 (11)	−0.026 (16)	−0.015 (14)
C32	0.093 (5)	0.059 (4)	0.079 (5)	−0.014 (4)	−0.032 (4)	−0.027 (4)
C33	0.088 (19)	0.06 (3)	0.07 (2)	−0.019 (19)	−0.028 (14)	−0.03 (2)
C34	0.090 (16)	0.063 (14)	0.070 (16)	−0.016 (11)	−0.026 (12)	−0.026 (8)
C35	0.084 (10)	0.066 (14)	0.071 (9)	−0.014 (11)	−0.022 (7)	−0.018 (11)
C36	0.090 (12)	0.065 (16)	0.071 (12)	−0.017 (10)	−0.027 (10)	−0.022 (10)
C33'	0.09 (4)	0.06 (6)	0.07 (5)	−0.02 (4)	−0.03 (3)	−0.03 (5)
C34'	0.09 (4)	0.06 (4)	0.07 (4)	−0.02 (3)	−0.03 (3)	−0.02 (2)
C35'	0.08 (3)	0.06 (4)	0.07 (3)	−0.01 (3)	−0.02 (2)	−0.02 (3)
C36'	0.09 (2)	0.07 (2)	0.07 (2)	−0.016 (17)	−0.027 (16)	−0.024 (17)
C37	0.065 (4)	0.055 (4)	0.069 (4)	−0.012 (3)	−0.007 (3)	−0.008 (3)
C38	0.062 (5)	0.082 (6)	0.141 (8)	0.003 (4)	−0.014 (5)	−0.018 (5)
C39	0.071 (4)	0.058 (4)	0.052 (4)	−0.025 (3)	−0.016 (3)	−0.004 (3)
C40	0.136 (7)	0.102 (6)	0.047 (4)	−0.054 (5)	−0.020 (4)	0.008 (4)

### Geometric parameters (Å, °)

**Table:**

Cu1—O4^i^	1.961 (3)	C12—H12B	0.9700
Cu1—O3	1.966 (3)	C13—C14	1.300 (9)
Cu1—O5	1.977 (3)	C14—C15	1.403 (10)
Cu1—O6^i^	1.989 (3)	C14—H14	0.9300
Cu1—N1	2.233 (4)	C15—C16	1.292 (10)
Cu1—Cu1^i^	2.7254 (11)	C15—H15	0.9300
Cu2—O10^ii^	1.959 (4)	C16—H16	0.9300
Cu2—O9	1.964 (4)	C17—C18	1.512 (7)
Cu2—O11	1.966 (4)	C18—H18A	0.9600
Cu2—O12^ii^	1.967 (4)	C18—H18B	0.9600
Cu2—N3	2.241 (4)	C18—H18C	0.9600
Cu2—Cu2^ii^	2.7085 (12)	C19—C20	1.508 (7)
N1—C1	1.320 (6)	C20—H20A	0.9600
N1—C3	1.401 (6)	C20—H20B	0.9600
N2—C1	1.371 (6)	C20—H20C	0.9600
N2—C2	1.377 (7)	C21—C28	1.437 (10)
N2—C12	1.469 (6)	C22—C27	1.392 (8)
N3—C21	1.321 (8)	C22—C23	1.404 (9)
N3—C27	1.398 (8)	C23—C24	1.330 (10)
N4—C22	1.355 (8)	C23—H23	0.9300
N4—C21	1.364 (8)	C24—C25	1.395 (10)
N4—C32	1.457 (7)	C24—H24	0.9300
O1—C11	1.368 (7)	C25—C26	1.381 (9)
O1—C8	1.378 (6)	C25—H25	0.9300
O2—C13	1.359 (8)	C26—C27	1.387 (9)
O2—C16	1.399 (8)	C26—H26	0.9300
O3—C17	1.247 (6)	C28—C29'	1.34 (3)
O4—C17	1.233 (6)	C28—C29	1.37 (2)
O4—Cu1^i^	1.961 (3)	C29—C30	1.38 (3)
O5—C19	1.248 (6)	C29—H29	0.9300
O6—C19	1.240 (6)	C30—C31	1.36 (4)
O6—Cu1^i^	1.989 (3)	C30—H30	0.9300
O7—C31	1.35 (3)	C31—H31	0.9300
O7—C28	1.371 (15)	C29'—C30'	1.34 (5)
O8—C36	1.35 (2)	C29'—H29'	0.9300
O8—C33	1.35 (9)	C30'—C31'	1.34 (5)
O7'—C31'	1.32 (3)	C30'—H30'	0.9300
O7'—C28	1.323 (15)	C31'—H31'	0.9300
O8'—C33'	1.32 (15)	C32—C33'	1.5 (2)
O8'—C36'	1.32 (3)	C32—C33	1.50 (10)
O9—C37	1.247 (7)	C32—H32A	0.9700
O10—C37	1.257 (7)	C32—H32B	0.9700
O10—Cu2^ii^	1.959 (4)	C32—H32C	0.9700
O11—C39	1.234 (7)	C32—H32D	0.9700
O12—C39	1.251 (7)	C33—C34	1.37 (10)
O12—Cu2^ii^	1.967 (4)	C34—C35	1.39 (5)
C1—C8	1.447 (7)	C34—H34	0.9300
C2—C3	1.377 (7)	C35—C36	1.37 (2)
C2—C7	1.396 (7)	C35—H35	0.9300
C3—C4	1.397 (7)	C36—H36	0.9300
C4—C5	1.375 (7)	C33'—C34'	1.3 (2)
C4—H4	0.9300	C34'—C35'	1.35 (10)
C5—C6	1.388 (8)	C34'—H34'	0.9300
C5—H5	0.9300	C35'—C36'	1.34 (5)
C6—C7	1.350 (8)	C35'—H35'	0.9300
C6—H6	0.9300	C36'—H36'	0.9300
C7—H7	0.9300	C37—C38	1.506 (9)
C8—C9	1.323 (7)	C38—H38A	0.9600
C9—C10	1.416 (8)	C38—H38B	0.9600
C9—H9	0.9300	C38—H38C	0.9600
C10—C11	1.298 (9)	C39—C40	1.505 (8)
C10—H10	0.9300	C40—H40A	0.9600
C11—H11	0.9300	C40—H40B	0.9600
C12—C13	1.486 (8)	C40—H40C	0.9600
C12—H12A	0.9700		
			
O4^i^—Cu1—O3	165.00 (14)	C19—C20—H20A	109.5
O4^i^—Cu1—O5	91.84 (16)	C19—C20—H20B	109.5
O3—Cu1—O5	86.28 (16)	H20A—C20—H20B	109.5
O4^i^—Cu1—O6^i^	87.21 (16)	C19—C20—H20C	109.5
O3—Cu1—O6^i^	90.80 (16)	H20A—C20—H20C	109.5
O5—Cu1—O6^i^	165.11 (15)	H20B—C20—H20C	109.5
O4^i^—Cu1—N1	99.23 (14)	N3—C21—N4	111.6 (6)
O3—Cu1—N1	95.76 (14)	N3—C21—C28	124.7 (6)
O5—Cu1—N1	98.95 (14)	N4—C21—C28	123.7 (6)
O6^i^—Cu1—N1	95.87 (14)	N4—C22—C27	107.3 (6)
O4^i^—Cu1—Cu1^i^	82.02 (10)	N4—C22—C23	133.0 (6)
O3—Cu1—Cu1^i^	82.99 (10)	C27—C22—C23	119.7 (7)
O5—Cu1—Cu1^i^	80.88 (10)	C24—C23—C22	118.4 (7)
O6^i^—Cu1—Cu1^i^	84.27 (11)	C24—C23—H23	120.8
N1—Cu1—Cu1^i^	178.75 (11)	C22—C23—H23	120.8
O10^ii^—Cu2—O9	165.48 (18)	C23—C24—C25	122.5 (7)
O10^ii^—Cu2—O11	87.25 (18)	C23—C24—H24	118.8
O9—Cu2—O11	88.85 (19)	C25—C24—H24	118.8
O10^ii^—Cu2—O12^ii^	92.30 (19)	C26—C25—C24	120.6 (7)
O9—Cu2—O12^ii^	87.95 (18)	C26—C25—H25	119.7
O11—Cu2—O12^ii^	165.31 (17)	C24—C25—H25	119.7
O10^ii^—Cu2—N3	99.22 (18)	C25—C26—C27	117.3 (7)
O9—Cu2—N3	95.11 (18)	C25—C26—H26	121.3
O11—Cu2—N3	96.54 (17)	C27—C26—H26	121.3
O12^ii^—Cu2—N3	98.02 (17)	C26—C27—C22	121.5 (6)
O10^ii^—Cu2—Cu2^ii^	80.40 (13)	C26—C27—N3	130.8 (5)
O9—Cu2—Cu2^ii^	85.37 (13)	C22—C27—N3	107.7 (6)
O11—Cu2—Cu2^ii^	85.86 (12)	O7'—C28—C29'	112.0 (16)
O12^ii^—Cu2—Cu2^ii^	79.60 (12)	O7'—C28—O7	112.9 (11)
N3—Cu2—Cu2^ii^	177.56 (13)	C29'—C28—O7	30.2 (9)
C1—N1—C3	104.9 (4)	O7'—C28—C29	37.5 (7)
C1—N1—Cu1	133.9 (3)	C29'—C28—C29	85.6 (15)
C3—N1—Cu1	121.2 (3)	O7—C28—C29	102.8 (11)
C1—N2—C2	106.3 (4)	O7'—C28—C21	116.4 (10)
C1—N2—C12	129.1 (5)	C29'—C28—C21	131.5 (14)
C2—N2—C12	124.2 (4)	O7—C28—C21	122.3 (9)
C21—N3—C27	106.2 (5)	C29—C28—C21	134.6 (10)
C21—N3—Cu2	134.3 (4)	C28—C29—C30	113.1 (16)
C27—N3—Cu2	119.5 (4)	C28—C29—H29	123.4
C22—N4—C21	107.3 (5)	C30—C29—H29	123.4
C22—N4—C32	123.1 (6)	C31—C30—C29	103 (3)
C21—N4—C32	129.5 (6)	C31—C30—H30	128.4
C11—O1—C8	104.9 (5)	C29—C30—H30	128.4
C13—O2—C16	104.8 (6)	O7—C31—C30	110 (2)
C17—O3—Cu1	123.4 (3)	O7—C31—H31	125.0
C17—O4—Cu1^i^	125.2 (3)	C30—C31—H31	125.0
C19—O5—Cu1	126.7 (3)	C28—C29'—C30'	106 (2)
C19—O6—Cu1^i^	122.0 (3)	C28—C29'—H29'	127.2
C31—O7—C28	110.3 (14)	C30'—C29'—H29'	127.2
C36—O8—C33	108 (4)	C31'—C30'—C29'	107 (3)
C31'—O7'—C28	104.3 (15)	C31'—C30'—H30'	126.6
C33'—O8'—C36'	107 (10)	C29'—C30'—H30'	126.6
C37—O9—Cu2	121.2 (4)	O7'—C31'—C30'	111 (3)
C37—O10—Cu2^ii^	127.4 (4)	O7'—C31'—H31'	124.4
C39—O11—Cu2	120.7 (4)	C30'—C31'—H31'	124.4
C39—O12—Cu2^ii^	127.9 (4)	N4—C32—C33'	115 (8)
N1—C1—N2	112.6 (5)	N4—C32—C33	106 (3)
N1—C1—C8	123.8 (4)	C33'—C32—C33	15 (5)
N2—C1—C8	123.7 (4)	N4—C32—H32A	110.5
C3—C2—N2	106.7 (4)	C33'—C32—H32A	116.2
C3—C2—C7	122.6 (5)	C33—C32—H32A	110.5
N2—C2—C7	130.7 (5)	N4—C32—H32B	110.5
C2—C3—C4	120.5 (5)	C33'—C32—H32B	95.3
C2—C3—N1	109.5 (4)	C33—C32—H32B	110.5
C4—C3—N1	130.1 (4)	H32A—C32—H32B	108.7
C5—C4—C3	116.6 (5)	N4—C32—H32C	108.9
C5—C4—H4	121.7	C33'—C32—H32C	108.8
C3—C4—H4	121.7	C33—C32—H32C	101.3
C4—C5—C6	121.7 (5)	H32A—C32—H32C	10.9
C4—C5—H5	119.1	H32B—C32—H32C	118.4
C6—C5—H5	119.1	N4—C32—H32D	108.5
C7—C6—C5	122.5 (5)	C33'—C32—H32D	108.5
C7—C6—H6	118.7	C33—C32—H32D	123.7
C5—C6—H6	118.7	H32A—C32—H32D	96.9
C6—C7—C2	116.1 (5)	H32B—C32—H32D	14.5
C6—C7—H7	121.9	H32C—C32—H32D	107.3
C2—C7—H7	121.9	O8—C33—C34	108 (7)
C9—C8—O1	110.2 (5)	O8—C33—C32	119 (6)
C9—C8—C1	133.1 (5)	C34—C33—C32	133 (7)
O1—C8—C1	116.6 (5)	C33—C34—C35	108 (4)
C8—C9—C10	106.4 (5)	C33—C34—H34	126.2
C8—C9—H9	126.8	C35—C34—H34	126.2
C10—C9—H9	126.8	C36—C35—C34	106 (3)
C11—C10—C9	107.4 (6)	C36—C35—H35	126.8
C11—C10—H10	126.3	C34—C35—H35	126.8
C9—C10—H10	126.3	O8—C36—C35	109 (2)
C10—C11—O1	111.1 (6)	O8—C36—H36	125.5
C10—C11—H11	124.4	C35—C36—H36	125.5
O1—C11—H11	124.4	O8'—C33'—C34'	110 (10)
N2—C12—C13	115.0 (5)	O8'—C33'—C32	121 (10)
N2—C12—H12A	108.5	C34'—C33'—C32	129 (10)
C13—C12—H12A	108.5	C33'—C34'—C35'	107 (9)
N2—C12—H12B	108.5	C33'—C34'—H34'	126.6
C13—C12—H12B	108.5	C35'—C34'—H34'	126.6
H12A—C12—H12B	107.5	C36'—C35'—C34'	107 (5)
C14—C13—O2	110.0 (6)	C36'—C35'—H35'	126.7
C14—C13—C12	135.0 (7)	C34'—C35'—H35'	126.7
O2—C13—C12	114.8 (6)	O8'—C36'—C35'	110 (3)
C13—C14—C15	108.3 (7)	O8'—C36'—H36'	125.2
C13—C14—H14	125.8	C35'—C36'—H36'	125.2
C15—C14—H14	125.8	O9—C37—O10	125.5 (6)
C16—C15—C14	106.8 (7)	O9—C37—C38	117.5 (6)
C16—C15—H15	126.6	O10—C37—C38	117.0 (6)
C14—C15—H15	126.6	C37—C38—H38A	109.5
C15—C16—O2	110.1 (7)	C37—C38—H38B	109.5
C15—C16—H16	124.9	H38A—C38—H38B	109.5
O2—C16—H16	124.9	C37—C38—H38C	109.5
O4—C17—O3	126.4 (5)	H38A—C38—H38C	109.5
O4—C17—C18	117.2 (5)	H38B—C38—H38C	109.5
O3—C17—C18	116.4 (5)	O11—C39—O12	125.9 (6)
C17—C18—H18A	109.5	O11—C39—C40	117.6 (6)
C17—C18—H18B	109.5	O12—C39—C40	116.5 (6)
H18A—C18—H18B	109.5	C39—C40—H40A	109.5
C17—C18—H18C	109.5	C39—C40—H40B	109.5
H18A—C18—H18C	109.5	H40A—C40—H40B	109.5
H18B—C18—H18C	109.5	C39—C40—H40C	109.5
O6—C19—O5	125.7 (5)	H40A—C40—H40C	109.5
O6—C19—C20	118.2 (5)	H40B—C40—H40C	109.5
O5—C19—C20	116.1 (5)		

Symmetry codes: (i) −*x*+2, −*y*+2, −*z*+1; (ii) −*x*+1, −*y*+1, −*z*.

### Hydrogen-bond geometry (Å, °)

**Table:**

*D*—H···*A*	*D*—H	H···*A*	*D*···*A*	*D*—H···*A*
C12—H12B···O3^iii^	0.97	2.55	3.426 (7)	151
C31—H31···O6^iv^	0.93	2.54	3.44 (3)	166
C36—H36···O4^i^	0.93	2.50	3.371 (19)	157

Symmetry codes: (iii) −*x*+2, −*y*+1, −*z*+1; (iv) −*x*+1, −*y*+2, −*z*+1; (i) −*x*+2, −*y*+2, −*z*+1.
